# Therapist-Assisted Web-Based Intervention for Prolonged Grief Disorder After Cancer Bereavement: Randomized Controlled Trial

**DOI:** 10.2196/27642

**Published:** 2022-02-08

**Authors:** Julia Kaiser, Michaela Nagl, Rahel Hoffmann, Katja Linde, Anette Kersting

**Affiliations:** 1 Department of Psychosomatic Medicine and Psychotherapy University of Leipzig Leipzig Germany

**Keywords:** digital interventions, grief, traumatic loss, treatment effectiveness evaluation, cognitive behavioral therapy, neoplasms

## Abstract

**Background:**

Bereavement due to cancer increases the risk of prolonged grief disorder. However, specialized treatment options for prolonged grief after a loss due to illness are still scarce.

**Objective:**

The aim of this study is to extend previous findings by evaluating a web-based cognitive behavioral intervention with asynchronous therapist support, consisting of structured writing tasks adapted specifically for prolonged grief after cancer bereavement.

**Methods:**

The intervention was evaluated in a purely web-based randomized waitlist-controlled trial. Open-access recruitment of participants was conducted on the web. Prolonged grief (Inventory of Complicated Grief), depression, anxiety, posttraumatic stress, posttraumatic growth, somatization, sleep quality, and mental and physical health were assessed on the web via validated self-report measures.

**Results:**

A total of 87 participants were randomized into the intervention group (IG; 44/87, 51%) or the waitlist control group (43/87, 49%). Of the participants, 7% (6/87) dropped out of the study (5/44, 11%, in the IG). Of the 39 completers in the IG, 37 (95%) completed all intervention tasks. The intervention reduced symptoms of prolonged grief (intention-to-treat: *P*<.001; *η*^2^=0.34; Cohen *d*=0.80) to a clinically significant extent. It had favorable effects on depression, anxiety, posttraumatic stress, posttraumatic growth, and overall mental health but not on somatization, sleep quality, or physical health.

**Conclusions:**

The web-based intervention for prolonged grief after cancer bereavement is effective in reducing symptoms of prolonged grief disorder and accompanying syndromes in a timely, easily realizable manner and addresses specific challenges of bereavement to illness. Considering web-based approaches in future mental health care policy and practice can reduce health care gaps for those who are bereaved to cancer.

**Trial Registration:**

German Clinical Trial Register U1111–1186-6255; https://www.drks.de/drks_web/navigate.do?navigationId=trial.HTML&TRIAL_ID=DRKS00011001

## Introduction

The loss of a loved one initiates a grief reaction, which is considered normal and healthy and enables adjustment to the loss and coping with new life realities. Although a normal grief reaction can be accompanied by significant emotional distress, the intensity of grief often decreases over a period that varies from culture to culture [1,2]. However, some bereaved persons show a grief reaction that is unusually long, intense, or complicated and can lead to significant impairment [2].

Pathological grief is included in the Diagnostic and Statistical Manual of Mental Disorders as persistent complex bereavement disorder (a diagnosis requiring further research) [3] and in the International Classification of Diseases (ICD-11) as prolonged grief disorder (PGD) [4]. With a conditional prevalence of 9.8% [5], PGD poses a considerable mental health risk for those who are bereaved of a loved one. The core symptoms of PGD include persistent and pervasive longing for or persistent preoccupation with the deceased and intense emotional pain, which may be reflected by sadness, anger, blame, guilt, numbness, the feeling of having lost a part of one’s self, and an inability to accept the loss, experience positive mood, or engage in social and other activities [4]. The grief reaction must persist for at least 6 months; exceed social, cultural, and religious norms for the bereaved person’s context; and cause significant impairment [4]. Persons with PGD report a reduction in quality of life, work and social functioning, energy levels, and overall mental health [6-8]. Risk factors for PGD include exposure to previous losses or trauma, previously impaired mental and physical health, low perceived social support, and low help-seeking [9,10].

A loss due to illness may cause specific additional strains for the bereaved, which increases the risk of developing PGD. Bereavement due to cancer has been identified as a risk factor for PGD [11,12]. Cancer is one of the leading causes of death in Germany. In 2016, a total of 229,827 persons died because of cancer, and 492,090 were diagnosed with cancer [13]. The diagnosis of a significant other is associated with a lower quality of life [14], increased distress [15,16], depression, and anxiety [17,18]. Such impairment of mental well-being during the time of illness was shown to predict further impairment of mental health in the case of bereavement [19,20]. Among bereaved caregivers, PGD is associated with preloss grief [20-23], preloss depression [20], the notion of not having coped well during the illness [23], poor family functioning [21], high caregiver burden [24], low preparedness, and low perceived social support [20,22]. A depletion of resources during the time of illness may impede bereavement adjustment [11,24] and put those who have a burdensome caregiving experience at increased risk of developing PGD after bereavement. A low perceived quality of death (agreement between preferences concerning the death and perceived actual circumstances of the death) [25], low preparedness for death, and death in a hospital [9,10] were further risk factors for PGD. The time of illness and the dying process, which are often experienced as burdensome or traumatic, influence the grieving process and should be specifically addressed in grief interventions.

Interventions targeting PGD have been proven effective [26]. Not all individuals with PGD, however, actually access treatment. Stigmatization [27] and low accessibility caused by conflicting schedules, long distances between client and therapist, or long waiting times may be barriers to treatment for the bereaved. Although caregiving and bereavement due to cancer pose a serious psychological strain, and studies examining interventions that specifically target cancer bereavement (eg, the studies by Kissane et al [28] and Lichtenthal et al [29]) have shown promising results, Guldin et al [30] reported that bereavement services in standard care do not target these aspects efficiently enough and therefore do not benefit those affected in a sufficient manner. This leaves bereaved relatives of persons with cancer at a high risk for adverse mental health outcomes, and their need for mental health care is often unmet.

Internet-based treatments offer an effective, flexible, and more anonymous approach for addressing mental health issues [31-33], which may help overcome treatment barriers for those with PGD. Internet-based treatment in general was shown to be as effective as conventional face-to-face treatment [34]. Internet-based interventions for grief have medium to large effect sizes [35]. Specific internet-based support for relatives of persons with cancer revealed promising results but, to date, mainly focused on caregiving during the time of illness (eg, the study by Applebaum et al [36]). There is a lack of evidence on internet-based interventions specifically addressing cancer bereavement and providing support beyond the time of illness. Web-based bereavement care targeted specifically at those with PGD after a cancer experience should be further examined.

Asynchronous web-based interventions that use cognitive behavioral techniques and rely on structured writing tasks and therapist feedback have proven effective in reducing syndromes such as prolonged grief, posttraumatic stress, or anxiety in the past (eg, the studies by Hedman et al [37] and Kersting et al [38]). They are often short and therefore economic and offer high flexibility for patients.

An asynchronous web-based intervention designed for the treatment of posttraumatic stress and PGD [39,40] has been successfully adapted by the research group to several specific bereavement situations such as pregnancy loss [38] or suicide bereavement [41]. To address the research gap concerning bereavement care after cancer, the intervention was adapted to suit the specific situation of those affected: difficult loss experiences are often preceded by a burdensome and possibly traumatic time of illness. The current intervention was designed to address the interlinking between grief and traumatic experiences, preloss grief and preparedness for the loss, and role conflicts and interpersonal conflicts. As a stand-alone, fully web-based intervention, it is suitable to overcome treatment barriers such as geographic and schedule restrictions and stigma. The effectiveness of the resulting therapist-assisted web-based intervention was evaluated in a randomized controlled trial to extend previous findings on bereavement care to the specific situation of cancer bereavement.

## Methods

### Study Design

The evaluation of the web-based cognitive behavioral therapy intervention for prolonged grief after bereavement due to cancer took place in a randomized waitlist-controlled trial. The primary outcome measure was prolonged grief. Prerandomization measurement points were screening (T-1) and baseline (T0), and postrandomization measurement points were posttreatment (T1) and follow-up (T2-T4).

The study was registered with the German Clinical Trial Register (Universal Trial Number U1111–1186-6255) and approved by the University of Leipzig Ethics Committee (no 450–15-21,122,015, January 20, 2017). The study was conducted in 2 waves with recruitment from November 2017 to April 2018 and from May 2018 to June 2019. The first wave was funded by *Deutsche José Carreras Leukämie-Stiftung* (German José Carreras Leukemia Foundation, DJCLS R15/22) and is thoroughly described in a study protocol [42]. The second wave followed the same methodology, except for more liberal inclusion criteria concerning the cause of bereavement, as specified in the next section. The recruitment duration of the first wave was determined by the duration of funding. The second wave was set to 1 year in advance.

### Participants

Individuals were eligible as participants if they:

- Were bereaved to hematological cancer (first wave) or any type of cancer (second wave),
- Reached a score of >25 on the Inventory of Complicated Grief (ICG) [43,44],
- Were ≥18 years, and
- Were fluent in the German language and had sufficiently stable web access.

The exclusion criteria were as follows:

- Current psychotherapy or change in psychopharmacological therapy within the last 6 weeks,
- Cognitive or physical impairment that would impede treatment participation, and
- Severe depression (Patient Health Questionnaire 9 [PHQ-9]; [45,46]), suicidal ideation (Beck Suicide Ideation Scale [47]; clinical assessment in telephone interview), dissociative tendency (Somatoform Dissociation Questionnaire [48]; clinical assessment in telephone interview), psychosis (Dutch Screening Device for Psychotic Disorder [49]; clinical assessment in telephone interview), posttraumatic stress disorder due to an event other than the loss (Impact of Event Scale–Revised [IES-R]; [50,51]), or substance use disorder [52].

### Procedure

Open-access recruitment was carried out from November 2017 to June 2019 via social networks, relevant websites, and stakeholders such as support groups, clinics, medical practices, charities, and insurance companies. Study information forms were presented on the website and again upon inclusion. Participants could apply for the study by taking the open-access web-based screening questionnaire (T-1) (see the study protocol by Hoffmann et al [42]). A subsequent telephone screening was carried out by the participant’s prospective therapist to clear any ambiguities concerning eligibility criteria (eg, to validate a possible positive screen for suicidality, psychosis, or dissociation) and to administer the Prolonged Grief Disorder Interview 13 [53,54] (not analyzed in this study). Informed consent was acquired as a signed form (mailed or scanned) from those who were included, and a baseline questionnaire (T0) was administered. Subsequently, randomization into either the intervention group (IG) or the waitlist control group (WCG) was conducted as described in the next section. Therefore, participants, as well as study personnel, were blinded to group allocation up to this point. After a treatment period of 5 weeks, a posttreatment measurement (T1) was administered to both groups. Afterward, participants in the WCG received the intervention and a second version of the posttreatment questionnaire (postintervention, T1.1). Follow-up measurements were administered at 3, 6, and 12 months after intervention completion. The entire study process was web based, except for 1 mandatory phone call per participant. All the data were stored in encrypted servers with password protection.

Measures to prevent multiple identities were informed consent forms, email confirmations, and phone calls. Participants did not pay for the intervention; neither were they paid.

**Figure 1:**
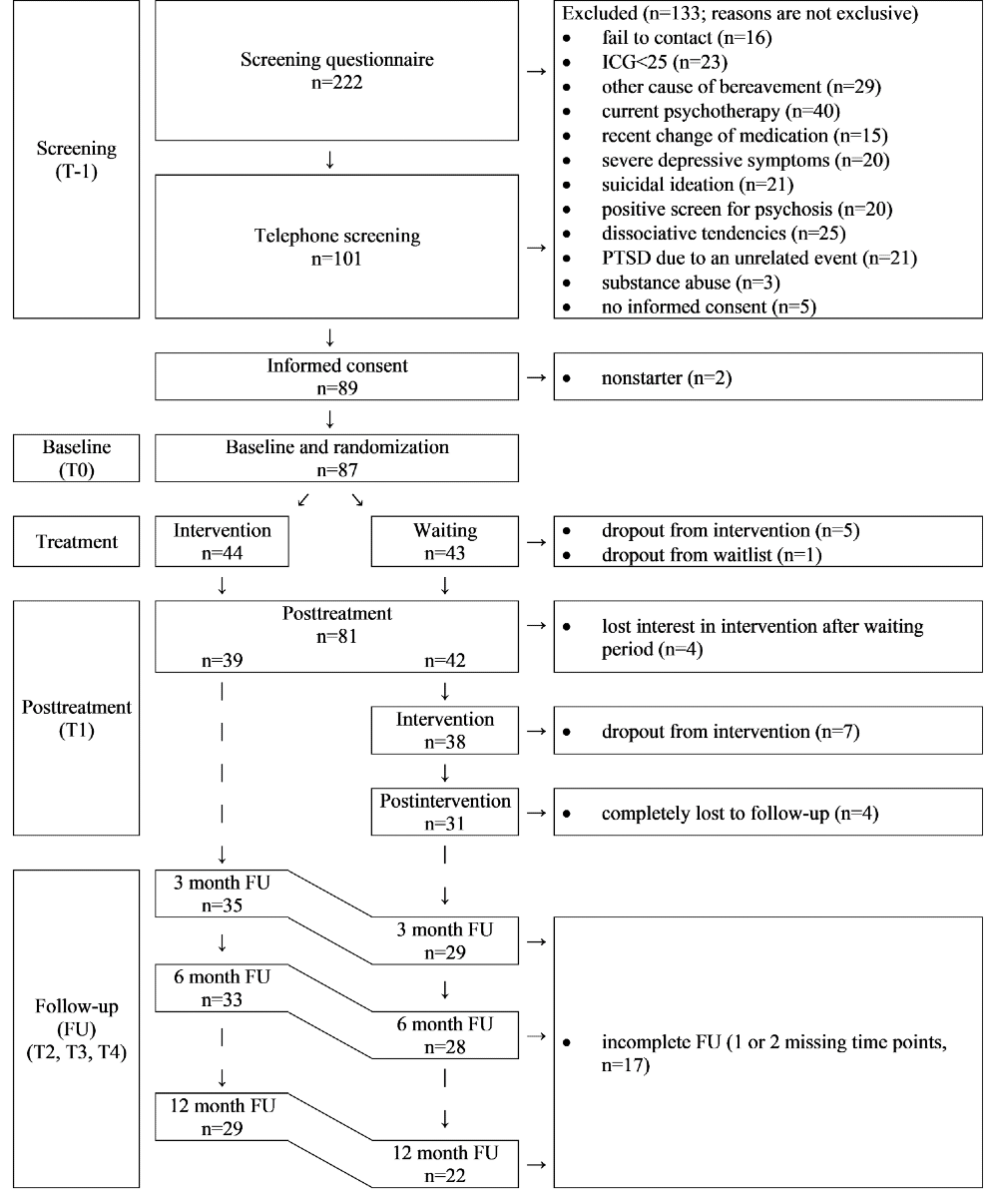
Participant flow. FU: follow-up; ICG: Inventory of Complicated Grief; PTSD: posttraumatic stress disorder.

The participant timeline is depicted in Figure 1.

### Randomization

Randomization was conducted with the software *Randomization in Treatment Arms* via permutated block randomization with a block size of 4 and equal probabilities to be sampled into either group (pseudoseeds, MersenneTwister). The allocation sequence was generated by a research assistant and stored separately from other study materials. Participants were automatically assigned to the treatment conditions after completing the baseline measurement.

### Intervention

The intervention *Online-Trauertherapie* (*Online Grief Therapy*) was conducted remotely via a secure website using the software *beranet* and consisted of 10 structured writing tasks that participants worked on independently in 2 self-scheduled 45-minute writing sessions per week. Participants received individualized therapist feedback from trained psychologists on all writing assignments within 24 hours, alternating between short and thorough feedback. If participants missed a scheduled session, they were reminded up to 2 times and called once if they did not respond. Participants could proactively contact their therapists via the website in case of questions or problems.

The structured writing tasks are organized into three modules (Table 1) that aim to work through grief and cope with the new situation: (1) *self-confrontation*, (2) *cognitive reappraisal*, and (3) *social sharing*.

**Table 1:** Intervention overview.

Phase and week			Procedure^a^				
			Pretask monitoring		Task		Posttask monitoring
**Phase 1: self-confrontation**							
	**1**						
		SAM^b^		1		SAM	
		SAM and PHQ-9^c^		2		SAM	
	**2**						
		SAM		3		SAM	
		SAM and PHQ-9		4		SAM and WAI-S^d^	
**Phase 2: cognitive reappraisal**							
	**3**						
		SAM		5		SAM	
		SAM and PHQ-9		6		SAM	
	**4**						
		SAM		7		SAM	
		SAM and PHQ-9		8		SAM and WAI-S	
**Phase 3: social sharing**							
	**5**						
		SAM		9		SAM	
		SAM and PHQ-9		10		SAM and WAI-S	

^a^At the end of every week, thorough therapist feedback was provided.

^b^SAM: Self-assessment Manikin.

^c^PHQ-9: Patient Health Questionnaire-9.

^d^WAI-S: Working Alliance Inventory–Short Form.

In the first module, *self-confrontation*, participants received information on prolonged grief, the treatment rationale, and the treatment platform. They were then asked to describe their loss experiences repeatedly in multiple sessions to reprocess traumatic memories and reduce avoidance behavior. Emphasis on emotional, cognitive, and bodily processes enables multimodal reprocessing. Addressed specifics of cancer bereavement included traumatic aspects of the illness experience, burdensome caregiving experiences, and ambiguity between hope for survival and preparation for the loss.

In the second module, *cognitive reappraisal*, participants were asked to write a letter to a hypothetical friend who shared the same loss experience. Participants were encouraged to focus on validating their counterparts’ suffering as well as on building and reinforcing resources and coping strategies. Specific to cancer bereavement was the validation of one’s own suffering, especially if the deceased’s suffering was so far perceived as more important, addressing of guilt and anger, and reflection of one’s role during the time of illness.

In the third module, *social sharing*, participants were asked to write (but not necessarily send) a letter to a real person involved in the loss experience (eg, themselves, the deceased, or a family member) to summarize and communicate their experiences, as well as new strategies and perspectives.

Instructions for all modules as well as psychoeducational material were standardized, and therapist feedback was highly structured but could be adapted to a specific patient’s situation.

The patient’s mood (Self-assessment Manikin [55]) and suicidality (PHQ-9 [45,46]) were monitored throughout the intervention to screen for increased distress and to provide information on emotional activation during the writing sessions. In addition, the Working Alliance Inventory–Short Form [56] was administered after every module. In case of increased distress, patients were contacted by their therapist via the platform or, if necessary, telephone.

### Measurements

#### Overview

A detailed account of all measurement tools can be found in Hoffmann et al [42]. All constructs examined in this study were assessed using web-based self-report questionnaires, except for Prolonged Grief Disorder Interview 13 (telephone). Sociodemographic variables and characteristics of the loss were examined before randomization (T-1 and T0). All outcomes were assessed at T0, T1, and all follow-up times. Consistency and completeness checks were conducted. Questionnaires had 24 to 48 pages, depending on the time point, with up to 15 items per page.

#### Primary Outcome

This study examines the primary outcome of prolonged grief, as measured using the German version of the ICG [43,44]. At T0, the ICG had an internal consistency of Cronbach α=.82.

#### Secondary Outcomes

The 19 original items of the ICG were augmented by 3 additional items adapted from Xiu et al [57] to fully capture the ICD-11 criteria of PGD. They address feelings of guilt, difficulty accessing positive memories, and anhedonia. The augmented version of the ICG with 22 items is considered a secondary outcome in all analyses (Cronbach α=.85).

Further secondary outcomes were depression (PHQ-9 [45,46]; Cronbach α=.86), anxiety (Generalized Anxiety Disorder Screener 7 [58,59]; Cronbach α=.84), posttraumatic stress due to the loss (IES-R [50,51]; Cronbach α=.85), posttraumatic growth (Posttraumatic Growth Inventory [60,61]; Cronbach α=.90), somatization (Patient Health Questionnaire-15 [45,46]; Cronbach α=.65), sleep quality (Pittsburgh Sleep Quality Index [62,63]; Cronbach α=.75), and physical and mental health (12-item Short-Form Health Survey [64,65]).

### Statistical Analyses

All statistical analyses were performed using R (R Foundation for Statistical Computing) [66]. Because both study waves were methodologically identical except for the cause of the bereavement inclusion criterion, a joint analysis was carried out for all participants across waves.

Descriptive analyses were used to provide means (SDs) or percentages of relevant variables. To test for baseline differences between treatment groups, between completers and dropouts, and between waves, 2-tailed *t* tests were used for numerical variables, and chi-square tests or the Fisher exact test for categorical variables.

The efficacy of the intervention was examined using linear mixed models for primary and secondary outcomes. This method allows for an intention-to-treat analysis under the assumption that data are missing at random. A restricted maximum-likelihood algorithm was applied. Analyses were performed in 2 steps. First, the appropriate base model was chosen by comparing the fit (Akaike information criterion and Bayesian information criterion) of three base models via analysis of variance: the unconditional means model (no random effects), random intercept model (unconditional growth model), and random intercept and random slope model. Second, fixed effects were added to the base model with the best fit to examine the effects of time, group, and time×group. Significance was assessed using *P* values approximated via Kenward–Roger approximations [67]. As there were 1 primary and 9 secondary outcomes, Bonferroni correction was applied, so that *P*=.05/10=.005 was deemed the threshold for significance in all linear mixed models. Effect sizes were calculated as Cohen *d* (between) and *η*^2^, with the latter representing the percentage of variance explained by the model. Effect sizes were considered small if Cohen *d*<0.5 (*η*^2^<.06), moderate if 0.5≤Cohen *d*<0.8 (0.06≤*η*^2^<0.14), and large if Cohen *d*≥0.8 (*η*^2^≥0.14) [68]. In addition, Hedges *g* was computed to aid comparability across studies. To explore within-group effects, separate multilevel analyses were performed for each of the 2 treatment groups for all outcomes with time as a fixed effect.

Additional analyses were carried out for the primary outcome (ICG) as follows:

1. In addition to the intention-to-treat analysis, a completer analysis was conducted using a linear mixed model.
2. To test for differences in symptom trajectories across waves, a separate linear mixed model was run with wave×time as an additional fixed effect.
3. The clinical significance of the change in ICG scores was evaluated using three metrics: (1) The reliable change index (RCI) [26] weighs pretest–posttest differences by SE (in this case, derived from Cronbach α at baseline). By comparing the score with *z* scores, a dichotomous assessment (95% CI) to determine whether a participant exhibited clinically significant improvement between 2 measurement points was derived. (2) The cut-off criterion of the ICG (25 points [43]) was used to provide an additional approximation of clinical significance, and (3) as a more conservative measure, the intersection of both was chosen, indicating clinically significant improvement only if both the RCI and cut-off criteria were satisfied. Chi-square tests were carried out for all 3 dichotomous measures (RCI, cut-off, and their intersection) to examine differences between treatment groups posttreatment.
4. An analysis of follow-up data (T2-T4) was conducted with a linear mixed model with time as a factor (postintervention vs 3-month follow-up vs 6-month follow-up vs 12-month follow-up). The model included both the treatment groups.

## Results

### Sample Description

A total of 222 persons completed the screening questionnaire, 89 (40.1%) of whom fulfilled the eligibility criteria and provided informed consent. The baseline questionnaire was completed by 87 participants, who were randomized into the IG (44/87, 51%) or the WCG (43/87, 49%). Participant flow is depicted in Figure 1.

Participants were on average 47.32 (SD 14.01) years old, and 83% (72/87) were female. Approximately half of the participants (42/87, 48%) were in a relationship, and 49% (43/87) had children (mean number of children, if any, 1.86, SD 1.17). Most participants had high (60/87, 69%) or intermediate (21/87, 24%) levels of education.

Participants were most often bereaved of their parents (41/87, 47%), spouses (30/87, 34%), or children (9/87, 10%), and reported a very close relationship with the deceased (mean 4.93, SD 0.30; on a scale of 1 [not close at all] to 5 [very close]). The death occurred on average 28.73 months (2.4 years) ago (SD 40.3, median 16.93 months, or 1.4 years). The most commonly reported cancer types among the deceased were leukemia (8/21, 38%) and lymphoma (6/21, 29%) in the first wave and cancer of the respiratory and chest organs (16/66, 24%) and digestive organs (13/66, 20%) in the second wave.

On average, participants reported an intensity of prolonged grief of mean_total_ 37.94 (SD_total_ 10.27; mean_IG_ 38.98, SD_IG_ 9.87; mean_WCG_ 36.88, SD_WCG_ 10.67; *P*=.35; on a scale of 0-76) at baseline.

The participants were assessed for secondary syndromes. Of all participants, 54% (47/87) scored above the threshold for at least moderate depression on the PHQ-9 (≥10), 39% (34/87) showed at least moderate anxiety (≥10), 17% (15/87) scored above the cut-off for likely posttraumatic stress disorder on the IES-R (>0), 44% (38/87) showed at least moderate somatization (≥10), and 32% (28/87) displayed severe sleep problems (>10). Overall, 76% (66/87) of the participants surpassed at least one of these thresholds. Of all participants, 86% (75/87) scored below the 20th percentile on the 12-item Short-Form Health Survey for mental health, whereas 35% (30/87) fell below the 20th percentile for physical health [69].

The treatment groups did not differ in sociodemographic variables, characteristics of the loss, or baseline mental health (Table 2). However, there was a significant difference in posttraumatic growth (*P=*.02), indicating that the IG reported significantly lower posttraumatic growth at baseline than did the WCG.

After randomization, 7% (6/87) of participants (5/44, 11% in the IG, 1/43, 2% in the WCG) dropped out of the study (ie, did not provide posttreatment data). Dropouts were exclusively female or nonbinary (*P=*.046) and reported slightly higher closeness to the deceased (mean_dropout_ 5.00 vs mean_completer_ 4.92; *P=*.03). Otherwise, there were no significant differences between completers and dropouts (Multimedia Appendix 1). Of the 39 completers in the IG, 37 (95%) completed all writing tasks, whereas 2 (5%) completed 7 and 9 tasks.

Participants recruited in the 2 waves did not differ, except for expected differences in the cause of loss (*P*<.001) and a smaller proportion of females among the deceased in the second wave (*P=*.01; Multimedia Appendix 1).

**Table 2:** Demographic and clinical characteristics of the study sample at baseline.

					Total (N=87)		Intervention group (n=44)		WCG^a^ (n=43)		*P* value^b^	
**Demographic characteristics**												
	Age (years), mean (SD)			47.32 (14.01)		47.80 (13.39)		46.84 (14.76)		.75		
	**Gender, n (%)**											.61
		Female		72 (83)		36 (82)		36 (84)				
		Male		14 (16)		7 (16)		7 (16)				
		Other		1 (1)		1 (2)		0 (0)				
	Relationship (yes), n (%)			42 (48)		19 (43)		23 (54)		.45		
	Has children (yes), n (%)			43 (49)		21 (47)		22 (51)		.92		
	Number of children (if any), mean (SD)			1.86 (1.17)		1.71 (0.9)		2 (1.38)		.43		
	**School education, n (%)**											.90
		Low		6 (7)		4 (9)		2 (5)				
		Intermediate		21 (24)		11 (25)		10 (23)				
		High		60 (69)		29 (66)		31 (69)				
**Characteristics of the loss**												
	Time since loss (months), mean (SD)			28.73 (40.3)		31.91 (50.65)		25.47 (26.02)		.46		
	**Relationship to the deceased, n (%)**											.86
		Parent		41 (47)		21 (48)		20 (47)				
		Child		9 (10)		3 (7)		6 (14)				
		Spouse		30 (35)		16 (36)		14 (33)				
		Sibling		3 (3)		2 (5)		1 (2)				
		Other		4 (5)		2 (5)		2 (5)				
	**Gender of the deceased, n (%)**											.45
		Female		43 (49)		24 (55)		19 (44)				
		Male		44 (51)		20 (45)		24 (56)				
		Other		0 (0)		0 (0)		0 (0)				
	Closeness to the deceased, mean (SD)			4.93 (0.30)		4.98 (0.15)		4.88 (0.39)		.15		
	**Type of cancer, n (%)**											.94
		**Hematological cancer**		31 (36)		17 (39)		14 (33)				
			Leukemia	10 (12)		5 (11)		5 (12)				
			Lymphoma	7 (8)		3 (7)		4 (9)				
			Plasmacytoma	6 (7)		4 (9)		2 (5)				
			Other hematological	8 (9)		5 (11)		3 (7)				
		**Other types of cancer**		56 (64)		27 (61)		29 (67)				
			Respiratory and chest organs	16 (18)		9 (21)		7 (16)				
			Digestive tract	13 (15)		5 (11)		8 (19)				
			Breast	6 (7)		2 (5)		4 (9)				
			Central nervous system and eyes	6 (7)		3 (7)		3 (7)				
			Urinary tract	3 (3)		1 (2)		2 (5)				
			Other	12 (14)		7 (16)		5 (12)				
**Mental health at baseline, mean (SD)**												
	Prolonged grief			37.94 (10.27)		38.98 (9.87)		36.88 (10.67)		.35		
	Depression			10.72 (5.33)		10.77 (5.08)		10.67 (5.63)		.93		
	Anxiety			8.39 (4.45)		8.68 (4.31)		8.09 (4.62)		.54		
	Posttraumatic stress			−0.83 (0.83)		−0.87 (0.83)		−0.80 (0.84)		.70		
	Posttraumatic growth			64.84 (18.13)		60.23 (18.3)		69.56 (16.89)		.02		
	Somatization			10.11 (4.51)		10.54 (4.66)		9.67 (4.36)		.37		
	Sleep quality			8.90 (3.71)		8.70 (3.54)		9.09 (3.91)		.62		
	Physical health			47.78 (10.13)		46.46 (10.66)		49.14 (9.5)		.22		
	Mental health			33.12 (9.44)		32.51 (8.63)		33.74 (10.26)		.55		

^a^WCG: waitlist control group.

^b^Group difference.

### Intervention Efficacy

#### Primary Outcome: Prolonged Grief

Baseline and posttreatment sum scores of prolonged grief measured with the ICG were used as outcomes of 3 base models (unconditional means, random intercept, random slope, and intercept), the fit of which was then compared via analysis of variance. A random intercept model provided the best fit (*P<*.001) and was used to examine the fixed effects of time, group, and the interaction of both (Table 3).

A significant group×time interaction effect indicated that prolonged grief decreased from baseline to posttreatment to a larger extent in the IG than in the WCG (*P<*.001; *F*_1,80.4_=40.7; N=87). The effect size was large (*η*^2^=0.34, 95% CI 0.20-0.46; Cohen *d*=0.80, 95% CI 0.35-1.25; Hedges *g*=0.79, 95% CI 0.34-1.24).

Separate random intercept models for each treatment group revealed a significant effect of time within the IG (*P<*.001; *F*_1,39.78_=58.89; N=44), but not within the WCG (*P=*.34; *F*_1, 41.11_=0.92; N=43).

A random intercept model with inclusion of completers only revealed results similar to the intention-to-treat analysis (time×group interaction: *P<*.001; *F*_1,79.2_=40.5), with large effect sizes (*η*^2^=0.34; Cohen *d*=0.80; N=81).

A random intercept model with the intention-to-treat sample and inclusion of wave×time as a fixed effect did not lead to an increase in model fit (*P=*.09), and the wave had no significant impact on the ICG score trajectory (wave×time interaction: *P=*.15; N=87).

According to the RCI, 44% (17/39) of the IG and 2% (1/42) of the WCG displayed clinically significant improvements in the ICG from baseline to posttreatment (*χ*^2^_1_=17.6; *P<*.001). The ICG cut-off of 25 was undercut by 44% (17/39) of the IG and 14% (6/42) of the WCG at posttreatment (*χ*^2^_1_=7.2; *P=*.007). Both criteria were met by 33% (13/39) in the IG and 2% (1/42) in the WCG (*χ*^2^_1_=11.5; *P<*.001).

Follow-up analysis showed that ICG scores directly after the intervention and at 3, 6, and 12 months after the intervention differed (*P<*.001; *F*_3,174.31_=6.48). Post hoc tests revealed that ICG scores were lower at follow-up (3 months vs postintervention, *P=*.009; 6 months vs postintervention, *P<*.001; 12 months vs postintervention, *P<*.001).

**Table 3:** Results of mixed model analyses (intention-to-treat, N=87).

Outcome				Pre, mean (SD)		Post, mean (SD)		Within-group effects of time				Interaction effects (time×group)										
								*F* test (*df*)		*P* value^a^		*F* test (*df*)			*P* value^a^			*η*^2^ (95% CI)			Cohen *d*_between_ (95% CI)	
**Primary outcome**																						
	**Prolonged grief (ICG^b^)**												40.7 (1,80.4)			<.001			0.34 (0.20 to 0.46)			0.80 (0.35 to 1.25)
		WCG^c^	36.9 (10.7)		36.0 (10.8)		0.9 (1,41.1)		.34													
		IG^d^	39.0 (9.9)		27.5 (10.4)		58.9 (1, 39.8)		<.001													
**Secondary outcomes**																						
	**Prolonged grief (ICGa^e^)**												44.4 (1,80.4)			<.001			0.36 (0.22 to 0.47)			0.84 (0.38 to 1.29)
		WCG	42.4 (12.3)		41.5 (12.6)		0.8 (1,41.1)		.38													
		IG	44.7 (11.5)		31.3 (11.9)		58.7 (1,39.6)		<.001													
	**Depression (PHQ-9^f^)**												21.0 (1,79.6)			<.001			0.21 (0.09 to 33)			0.69 (0.23 to 1.13)
		WCG	10.7 (5.6)		9.4 (4.8)		4.4 (1,40.2)		.04													
		IG	10.8 (5.1)		6.4 (3.9)		44.7 (1,39.8)		<.001													
	**Anxiety (GAD-7^g^)**												8.7 (1,80.4)			.004			0.10 (0.02 to 0.21)			0.43 (−0.01 to 0.88)
		WCG	8.1 (4.6)		7.4 (3.9)		0.8 (1,40.5)		.39													
		IG	8.7 (4.3)		5.9 (3.1)		20.6 (1,40.3)		<.001													
	**Posttraumatic stress (IES-R^h^)**												9.1 (1,80.4)			.003			0.10 (0.02 to 0.22)			0.65 (0.20 to 1.10)
		WCG	−0.8 (0.8)		−1.0 (0.8)		5.4 (1,40.3)		.03													
		IG	−0.9 (0.8)		−1.6 (0.8)		21.1 (1,40.6)		<.001													
	**Posttraumatic growth (PGI^i^)**												24.6 (1,79.7)			<.001			0.24 (0.11 to 0.36)			−0.29 (−0.73 to 0.15)
		WCG	69.6 (16.9)		70.6 (16.7)		0.1 (1,40.4)		.75													
		IG	60.2 (18.3)		76.1 (21.0)		42.4 (1,39.2)		<.001													
	**Somatization (PHQ-15^j^)**												1.9 (1,79.4)			.17			0.02 (0.00 to 10)			−0.03 (−0.47 to 0.41)
		WCG	9.7 (4.4)		8.5 (3.7)		4.9 (1,40.4)		.03													
		IG	10.5 (4.7)		8.6 (4.8)		13.2 (1,39.0)		<.001													
	**Sleep quality (PSQI^k^)**												0.02 (1,77.7)			.90			0.00 (0.00 to 0.02)			−0.01 (−0.46 to 0.43)
		WCG	9.1 (3.9)		8.5 (3.6)		0.7 (1,39.7)		.41													
		IG	8.7 (3.5)		8.6 (3.8)		0.3 (1,38.1)		.58													
	**Physical health (SF-12^l^)**												0.1 (1,79.6)			.77			0.00 (0.00 to 0.04)			0.23 (−0.21 to 0.67)
		WCG	49.1 (9.5)		49.5 (8.1)		0.0 (1,40.4)		.95													
		IG	46.5 (10.7)		47.5 (9.7)		0.2 (1,39.2)		.66													
	**Mental health (SF-12)**												8.6 (1,80.8)			.004			0.10 (0.02 to 0.21)			−0.44 (−0.89 to 0.00)
		WCG	33.7 (10.3)		34.8 (11.3)		0.4 (1,40.4)		.53													
		IG	32.5 (8.6)		39.3 (8.8)		15.4 (1,41.2)		<.001													

^a^Values of *P*<.005 were considered to indicate significance.

^b^ICG: Inventory of Complicated Grief.

^c^WCG: waitlist control group.

^d^IG: intervention group.

^e^ICGa: augmented version of Inventory of Complicated Grief.

^f^PHQ-9: Patient Health Questionnaire-9.

^g^GAD-7: Generalized Anxiety Disorder-7 scale.

^h^IES-R: Impact of Event Scale–Revised.

^i^PGI: Posttraumatic Growth Inventory.

^j^PHQ-15: Patient Health Questionnaire-15.

^k^PSQI: Pittsburgh Sleep Quality Index.

^l^SF-12: 12-item Short Form Health Survey.

#### Secondary Outcomes

Prolonged grief as measured with the augmented version of the ICG, depression, anxiety, posttraumatic stress, somatization, mental and physical health, sleep quality, and posttraumatic growth were examined as secondary outcomes (Table 3). A random intercept model provided the best fit for all secondary outcomes. A significant group×time interaction was found for prolonged grief (augmented), depression, anxiety, posttraumatic stress, posttraumatic growth, and mental health with effect sizes from Cohen *d*=0.29 to 0.84 (small to large), but not for physical health, sleep quality, or somatization. A significant within-group effect of time was found in the IG for prolonged grief (augmented), depression, anxiety, posttraumatic stress, posttraumatic growth, mental health, and somatization and in the WCG for depression, posttraumatic stress, and somatization (Table 3). There was no deterioration in the mean scores of any secondary outcome. No unintended effects were observed.

## Discussion

### Principal Findings

In light of unmet mental health care needs among those bereaved by cancer, we adapted and evaluated a web-based intervention for PGD after cancer bereavement. Specifically, the intervention was designed to address the traumatic nature of the time of illness as well as difficulties in the bereavement phase. It exceeds the scope of previously evaluated web-based interventions for relatives of patients with cancer. The intervention proved effective in reducing symptoms of PGD to a clinically significant extent compared with a WCG.

A total of 87 participants were included and randomized. With 6 participants dropping out, 81 completed the posttreatment measurement. The dropout rate of 7% is in line with previous studies on web-based interventions for grief [35], which indicates sufficient acceptability.

With 76% of participants exceeding cut-offs for at least one secondary syndrome, and 86% scoring below the 20th percentile for mental health, our sample displayed considerable impairment before treatment, which illustrates the necessity of an accessible intervention.

A linear mixed model was used to examine the intervention’s efficacy and revealed a significant interaction effect, indicating a greater decrease in PGD symptoms (ICG) in the IG than in the WCG. This effect proved robust in a completer analysis and in an analysis including the augmented version of the ICG with 3 additional items that reflect specifics of the ICD-11 criteria [57]. The intervention had a large effect on PGD symptoms (Cohen *d*=0.80; Hedges *g*=0.79) and led to clinically significant improvement. The effect size in this study exceeded the average pooled effect sizes from two recent meta-analyses examining (1) conventional and web-based interventions for prolonged grief (Hedges *g*=0.45) [26] and (2) only web-based grief interventions (Hedges *g*=0.54, 95% CI 0.30-0.78) [35]. Symptoms of PGD further decreased throughout the follow-up period of 12 months. These results indicate that the intervention is suitable for decreasing the symptoms of PGD to a relevant extent.

Small to moderate effects were found for depression, anxiety, posttraumatic stress, posttraumatic growth, and mental health. This shows that the intervention is suitable not only to decrease PGD but also to ameliorate accompanying syndromes and overall mental health. Some modules of the intervention are well suited to address syndromes besides PGD. Especially, (1) the module *self-confrontation* facilitates reprocessing of distressing memories and may therefore lead to a decrease in posttraumatic stress and related anxiety, and (2) the module *cognitive reappraisal* is set to improve coping skills and resource availability and may therefore influence depressive symptoms and posttraumatic growth. The effect size for depression in this study was comparable with the pooled effect size found by Wagner et al [35] for web-based grief interventions; the effect size for posttraumatic stress was slightly lower. The absence of an effect on physical health, somatization, and sleep is deemed conclusive, because these constructs are related to physical well-being, which was not targeted in the intervention.

We argue that this study is methodologically suitable for examining the effectiveness of a web-based intervention for PGD. However, some methodological aspects merit discussion.

As stated in the study protocol [42], a sample size of N=128 was intended to ensure enough power to detect a moderate effect. Although we did not meet this criterion, the achieved sample size of N=87 was sufficient to detect the large effect that the intervention had on PGD.

This study was conducted in 2 waves, with the second wave’s (May 2018 to June 2019) inclusion criteria concerning the cause of bereavement being more liberal than the first wave’s (November 2017 to April 2018). However, participants of both waves displayed similar amounts of distress, were from similar socioeconomic backgrounds, and had similar characteristics of their loss. Moreover, the PGD trajectories did not differ between the waves. Therefore, we deemed the groups homogeneous enough to be included in the joint analysis.

Treatment groups were considered mostly equal, as they differed only in that the WCG had more favorable values for posttraumatic growth at baseline than the IG. This may, to some extent, weaken the interpretability of the results concerning posttraumatic growth.

Females were overrepresented in this study, as is the case in many previous studies on web-based interventions [35] or caregiving and bereavement [24]. To some extent, this may reflect women in Germany being more often affected by bereavement than men. For example, women are more often widowed than men [70]. In addition, women do more often assume caregiver roles for sick relatives [71], which makes them more vulnerable to burdensome caregiving experiences and witnessing traumatic aspects of illness and death. This may lead to increased PGD levels among women compared with men. However, women may also be more likely to seek support via the internet or be open to therapist contact [72]. Therefore, our sample, which is not representative of the German population, may well be representative of those who have PGD after a loss due to illness and are willing to undergo web-based treatment.

This study relied on web-based self-report measures to assess the primary and secondary outcomes. Although the use of interviews would have provided added validity, our questionnaires comprised instruments that were designed and validated for administration as self-report assessments. Therefore, we deemed our assessments to be adequately valid.

Future research may examine the differential effects of the treatment modules used in this study, the role of therapist support, and the long-term effects of web-based interventions, especially in comparison with face-to-face approaches. In addition, it might be fruitful to explore the acceptability and effectiveness of web-based grief interventions when blended into existing health care structures (eg, primary care) and to examine economic aspects such as cost-effectiveness.

### Conclusions

PGD has significant ramifications for individuals and society. As it has only recently been acknowledged as a mental illness, specialized treatment options are still scarce. A low-threshold, acceptable, and effective web-based intervention may reduce treatment barriers and improve the mental health care situation of those affected.

Our results extend previous findings by providing evidence for the efficacy of a web-based intervention that was specifically adapted for persons bereaved because of cancer. It proved effective in decreasing the symptoms of PGD and accompanying syndromes to a clinically significant extent in a relatively short treatment duration of 5 weeks. It addresses specific issues of cancer bereavement, such as traumatic aspects of the time of illness, preloss grief, and preparedness, and provides low-threshold access to specialized grief therapy. Therefore, it is suitable to reduce the treatment gap for those with PGD after a loss due to illness.

Alternatives and complements to conventional face-to-face psychotherapy are needed, as illustrated by the increased demand for remote treatment options during the COVID-19-pandemic. Web-based approaches should therefore be considered in future mental health care policies and practices.

## Appendix

Multimedia Appendix 1 Demographic and clinical characteristics of the study sample at baseline by dropout status and study wave.

Multimedia Appendix 2 CONSORT-eHEALTH checklist (V 1.6.1).

## Abbreviations

ICD-11: International Classification of Diseases
ICG: Inventory of Complicated Grief
IES-R: Impact of Event Scale–Revised
IG: intervention group
PGD: prolonged grief disorder
PHQ-9: Patient Health Questionnaire 9
RCI: reliable change index
WCG: waitlist control group
