# Supplementary material for: Therapist-Assisted Web-Based Intervention for Prolonged Grief Disorder After Cancer Bereavement: Randomized Controlled Trial
Source: JMIR Ment Health. 2022 Feb 8;9(2):e27642. doi: 10.2196/27642 (PMC8864524; doi:10.2196/27642)
Supplement: Multimedia Appendix 1 [file mental_v9i2e27642_app1.docx]

Multimedia Appendix 1.A Comparison of baseline characteristics between completers and dropouts.

|  |  | | **Total**  **(N = 87)** | | **Completer**  **(N = 81)** | | **Dropout**  **(N = 6)** | | **Group Difference** |
| --- | --- | --- | --- | --- | --- | --- | --- | --- | --- |
|  |  | | M/N | (SD)/(%) | M/N | (SD)/(%) | M/N | (SD)/(%) | **p** |
| ***Methodological variables*** |  | |  |  |  |  |  |  |  |
|  | **Group allocation** | IG | 44 | 50.6 | 39 | 48.1 | 5 | 83.3 | 0.215 |
| ***Demographic Characteristics*** |  | |  |  |  |  |  |  |  |
|  | **Age** |  | 47.32 | (14.01) | 47.2 | (14.15) | 49 | (12.93) | .763 |
|  | **Gender** |  |  |  |  |  |  |  |  |
|  |  | Female | 72 | (82.8%) | 67 | (82.7%) | 5 | (83.3%) | .046 |
|  |  | Male | 14 | (16.1%) | 14 | (17.3%) | 0 | (0.0%) |  |
|  |  | Other | 1 | (1.1%) | 0 | (0.0%) | 1 | (16.7%) |  |
|  | **Relationship** | Yes | 42 | (48.3%) | 38 | (46.9%) | 4 | (66.7%) | .423 |
|  | **Has Children** | Yes | 43 | (49.4%) | 38 | (46.9%) | 5 | (83.3%) | .194 |
|  | **Number of Children, if any** | | 1.86 | (1.17) | 1.89 | (1.2) | 1.6 | (0.89) | .601 |
|  | **School education** | |  |  |  |  |  |  |  |
|  |  | low | 6 | (6.9%) | 4 | (4.9%) | 2 | (33.3%) | .081 |
|  |  | intermediate | 21 | (24.1%) | 20 | (24.7%) | 1 | (16.7%) |  |
|  |  | high | 60 | 69.0 | 57 | (70.4%) | 3 | (50.0%) |  |
| ***Characteristics of the Loss*** |  | |  |  |  |  |  |  |  |
|  | **Time since loss (months)** | | 28.73 | (40.30) | 28.49 | (40.94) | 31.91 | (33.15) | .843 |
|  | **Relationship to the deceased** | |  |  |  |  |  |  | .131 |
|  |  | Parent | 41 | (47.1%) | 39 | (48.1%) | 2 | (33.3%) |  |
|  |  | Child | 9 | (10.3%) | 7 | (8.6%) | 2 | (33.3%) |  |
|  |  | Spouse | 30 | (34.5%) | 29 | (35.8%) | 1 | (16.7%) |  |
|  |  | Sibling | 3 | (3.4%) | 3 | (3.7%) | 0 | (0.0%) |  |
|  |  | Other | 4 | (4.6%) | 3 | (3.7%) | 1 | (16.7%) |  |
|  | **Gender of the deceased** | |  |  |  |  |  |  | > .999 |
|  |  | Female | 43 | (49.4%) | 40 | (49.4%) | 3 | (50%) |  |
|  |  | Male | 44 | (50.6%) | 41 | (50.6%) | 3 | (50%) |  |
|  |  | Other | 0 | (0.0%) | 0 | (0.0%) | 0 | (0.0%) |  |
|  | **Closeness to the deceased** | | 4.93 | (0.3) | 4.93 | (0.31) | 5 | (0) | .033^a^ |
|  | **Type of cancer** | |  |  |  |  |  |  |  |
|  | *Hematological cancer* | |  |  |  |  |  |  | .571 |
|  | Leukemia | | 10 | (11.5%) | 9 | (11.1%) | 1 | (16.7%) |  |
|  | Lymphoma | | 7 | (8.0%) | 6 | (7.4%) | 1 | (16.7%) |  |
|  | Plasmocytoma | | 6 | (6.9%) | 6 | (7.4%) | 0 | (0.0%) |  |
|  | Other hematological | | 8 | (9.2%) | 6 | (7.4%) | 2 | (33.3%) |  |
|  | *Other types of cancer* | |  |  |  |  |  |  |  |
|  | Respiratory and chest organs | | 16 | (18.4%) | 16 | (19.8%) | 0 | (0.0%) |  |
|  | Digestive tract | | 13 | (14.9%) | 12 | (14.8%) | 1 | (16.7%) |  |
|  | Breast | | 6 | (6.9%) | 6 | (7.4%) | 0 | (0.0%) |  |
|  | Central nervous system & eyes | | 6 | (6.9%) | 6 | (7.4%) | 0 | (0.0%) |  |
|  | Urinary tract | | 3 | (3.4%) | 3 | (3.7%) | 0 | (0.0%) |  |
|  | Other | | 12 | (13.8%) | 11 | (13.6%) | 1 | (16.7%) |  |
| ***Mental Health at Baseline*** |  | |  |  |  |  |  |  |  |
|  | **Prolonged grief** | | 37.94 | (10.27) | 37.79 | (10.33) | 40.00 | (10.16) | .614 |
|  | **Depression** | | 10.72 | (5.33) | 10.75 | (5.30) | 10.33 | (6.25) | .853 |
|  | **Somatization** | | 10.11 | (4.51) | 10.05 | (4.59) | 10.89 | (3.55) | .662 |
|  | **Posttraumatic stress** | | -0.83 | (0.83) | -0.83 | (0.85) | -0.83 | (0.66) | .995 |
|  | **Anxiety** | | 8.39 | (4.45) | 8.42 | (4.55) | 8.00 | (3.03) | .825 |
|  | **Sleep Quality** | | 8.90 | (3.71) | 8.90 | (3.78) | 8.83 | (2.93) | .966 |
|  | **Posttraumatic Growth** | | 64.84 | (18.13) | 65.48 | (18.05) | 56.17 | (18.52) | .227 |
|  | **Physical Health** | | 47.78 | (10.13) | 48.12 | (10.32) | 43.20 | (5.74) | .253 |
|  | **Mental Health** | | 33.12 | (9.44) | 33.17 | (9.63) | 32.34 | (6.82) | .836 |
| ^a^due to nonequal variances, the Welch two sample t-test was used. | | | | | | | | | |

Multimedia Appendix 1.B Comparison of baseline characteristics between waves.

|  |  | | **Total**  **(N = 87)** | | **Wave 1**  **(N = 21)** | | **Wave 2**  **(N = 66)** | | **Group Difference** |
| --- | --- | --- | --- | --- | --- | --- | --- | --- | --- |
|  |  | | M/N | (SD)/(%) | M/N | (SD)/(%) | M/N | (SD)/(%) | **p** |
| ***Demographic Characteristics*** |  | |  |  |  |  |  |  |  |
|  | **Age** |  | 47.32 | (14.01) | 47.52 | (13.12) | 47.26 | (14.38) | .940 |
|  | **Gender** |  |  |  |  |  |  |  | .201 |
|  |  | Female | 72 | (82.8%) | 17 | (81.0%) | 55 | (83.3%) |  |
|  |  | Male | 14 | (16.1%) | 3 | (14.3%) | 11 | (16.7%) |  |
|  |  | Other | 1 | (1.1%) | 1 | (4.8%) | 0 | (0.0%) |  |
|  | **In a Relationship** | Yes | 42 | (48.3%) | 10 | (47.6%) | 32 | (48.5%) | > .999 |
|  | **Has Children** | Yes | 43 | (49.4%) | 9 | (42.9%) | 34 | (51.5%) | .618 |
|  | **Number of Children, if any** | | 1.86 | (1.17) | 2.11 | (1.62) | 1.79 | (1.04) | .475 |
|  | **School education** |  |  |  |  |  |  |  | .075 |
|  |  | low | 6 | (6.9%) | 1 | (4.8%) | 5 | (7.6%) |  |
|  |  | intermediate | 21 | (24.1%) | 9 | (42.9%) | 12 | (18.2%) |  |
|  |  | high | 60 | (69.0%) | 11 | (52.4%) | 49 | (74.2%) |  |
| ***Characteristics of the Loss*** |  | |  |  |  |  |  |  |  |
|  | **Time since loss (months)** | | 28.73 | (40.3) | 31.62 | (29.02) | 27.81 | (43.43) | .708 |
|  | **Relationship to the deceased** | |  |  |  |  |  |  | .933 |
|  |  | Parent | 41 | (47.1%) | 10 | (47.6%) | 31 | (47.0%) |  |
|  |  | Child | 9 | (10.3%) | 3 | (14.3%) | 6 | (9.1%) |  |
|  |  | Spouse | 30 | (34.5%) | 6 | (28.6%) | 24 | (36.4%) |  |
|  |  | Sibling | 3 | (3.4%) | 1 | (4.8%) | 2 | (3.0%) |  |
|  |  | Other | 4 | (4.6%) | 1 | (4.8%) | 3 | (4.5%) |  |
|  | **Gender of the deceased** | |  |  |  |  |  |  | .014 |
|  |  | Female | 43 | (49.4%) | 5 | (23.8%) | 38 | (57.6%) |  |
|  |  | Male | 44 | (50.6%) | 16 | (76.2%) | 28 | (42.4%) |  |
|  |  | Other | 0 | (0.0%) | 0 | (0.0%) | 0 | (0.0%) |  |
|  | **Closeness to the deceased** | | 4.93 | (0.3) | 5 | (0) | 4.91 | (0.34) | .224 |
|  | **Type of cancer** | |  |  |  |  |  |  |  |
|  | *Hematological cancer* | |  |  |  |  |  |  | < .001 |
|  | Leukemia | | 10 | (11.5%) | 8 | (38.1%) | 2 | (3.0%) |  |
|  | Lymphoma | | 7 | (8.0%) | 6 | (28.6%) | 1 | (1.5%) |  |
|  | Plasmocytoma | | 6 | (6.9%) | 2 | (9.5%) | 4 | (6.1%) |  |
|  | Other hematological | | 8 | (9.2%) | 5 | (23.8%) | 3 | (4.5%) |  |
|  | *Other types of cancer* | |  |  |  |  |  |  |  |
|  | Respiratory and chest organs | | 16 | (18.4%) | 0 | (0.0%) | 16 | (24.2%) |  |
|  | Digestive tract | | 13 | (14.9%) | 0 | (0.0%) | 13 | (19.7%) |  |
|  | Breast | | 6 | (6.9%) | 0 | (0.0%) | 6 | (9.1%) |  |
|  | Central nervous system & eyes | | 6 | (6.9%) | 0 | (0.0%) | 6 | (9.1%) |  |
|  | Urinary tract | | 3 | (3.4%) | 0 | (0.0%) | 3 | (4.5%) |  |
|  | Other | | 12 | (13.8%) | 0 | (0.0%) | 12 | (18.2%) |  |
| ***Mental Health at Baseline*** |  | |  |  |  |  |  |  |  |
|  | **Prolonged grief** (ICG) | | 37.94 | (10.27 ) | 36.05 | ( 9.87) | 38.55 | ( 10.40) | .335 |
|  | **Depression** (PHQ9) | | 10.72 | (5.33) | 10.05 | (5.91) | 10.94 | (5.16) | .507 |
|  | **Somatization** (PHQ15) | | 10.11 | (4.51) | 10.20 | (4.10) | 10.08 | (4.66) | .914 |
|  | **Posttraumatic stress** (IESR) | | -0.83 | (0.83) | -1.09 | (0.75) | -0.75 | (0.84) | .101 |
|  | **Anxiety** (GAD) | | 8.39 | (4.45) | 7.05 | (4.63) | 8.82 | (4.34) | .113 |
|  | **Sleep Quality** (PSQI) | | 8.90 | (3.71) | 8.40 | (4.35) | 9.05 | (3.52) | .499 |
|  | **Posttraumatic Growth** (PGI) | | 64.84 | (18.13) | 70.76 | (16.15) | 62.95 | (18.43) | .086 |
|  | **Physical Health** (SF-12) | | 47.78 | (10.13) | 48.16 | (9.50) | 47.67 | (10.39) | .847 |
|  | **Mental Health** (SF-12) | | 33.12 | (9.44) | 35.23 | (10.68) | 32.44 | (8.99) | .240 |
|  |  | |  |  |  |  |  |  |  |
